# Bayesian Methods to Analyze Historical Collections in Time and Space: A Case Study Using Cabo Verde Endemic Flora

**DOI:** 10.3389/fpls.2020.00278

**Published:** 2020-03-13

**Authors:** Maria M. Romeiras, Mark Carine, Maria Cristina Duarte, Silvia Catarino, Filipe S. Dias, Luís Borda-de-Água

**Affiliations:** ^1^LEAF, Linking Landscape, Environment, Agriculture and Food, Instituto Superior de Agronomia (ISA), Universidade de Lisboa, Lisbon, Portugal; ^2^Centre for Ecology, Evolution and Environmental Changes (cE3c), Faculdade de Ciências, Universidade de Lisboa, Campo Grande, Portugal; ^3^Department of Life Sciences, The Natural History Museum, London, United Kingdom; ^4^CIBIO/InBio, Centro de Investigação em Biodiversidade e Recursos Genéticos, Laboratório Associado, Universidade do Porto, Campus Agrário de Vairão, Vairão, Portugal; ^5^CIBIO/InBio, Centro de Investigação em Biodiversidade e Recursos Genéticos, Laboratório Associado, ISA, Universidade de Lisboa, Lisbon, Portugal

**Keywords:** Bayesian methods, Gaussian processes, Macaronesian islands, scientific expeditions, species discovery, type specimens

## Abstract

Biological collections, including herbarium specimens, are unique sources of biodiversity data presenting a window on the history of the development and accumulation of knowledge of a specific geographical region. Understanding how the process of discovery impacts that knowledge is particularly important for oceanic islands which are often characterized by both high levels of endemic diversity and high proportions of threatened taxa. The archipelagos of the Macaronesian region (i.e. Azores, Canaries, Savages, Madeira, and Cabo Verde) have been the focus of attention for scientific expeditions since the end of the 17th century. However, there is no integrated study describing the historical process of collecting, discovery and description of its flora. Using as a case study the Cabo Verde endemic angiosperm flora, we review the history of collecting in the flora and apply a Bayesian approach to assess the accumulation of species discovery, through time and space across the nine islands of the archipelago. Our results highlight the central role not only of natural characteristics (e.g. area, age, maximum altitude and average value of the terrain ruggedness index) but also historical factors (i.e. the location of major harbors) for the development of knowledge of the flora. The main factors that have determined the process of species description in the archipelago and how this impact our understanding of diversity patterns across archipelagos are discussed.

## Introduction

Understanding global patterns of plant diversity is particularly important for oceanic islands, which are often characterized by high levels of endemic diversity (Warren et al., 2015; Whittaker et al., 2017). The Macaronesian region has been recognized as a biogeographically related group of five volcanic archipelagos (i.e. Azores, Madeira, Savage, Canary, and Cabo Verde Islands) and is renowned for its biodiversity thanks to their extraordinary levels of species diversity and endemism (e.g. Caujapé-Castells et al., 2017). The concentration of endemic plants in Macaronesia reveals the importance of baseline data on taxonomy and species distribution, as only these can provide the tools needed for their conservation (e.g. Romeiras et al., 2016). Despite the long-standing interest in the Macaronesian flora, there is no complete flora for the region and the existing literature is heterogeneous. Among the most important recent initiatives has been the publication of checklists for each archipelago (e.g. Cabo Verde: Arechavaleta et al., 2005; Madeira and Salvages: Borges et al., 2008; Azores: Borges et al., 2010; Canaries: Arechavaleta et al., 2010). These provide important data for research on biodiversity, but there is still incomplete knowledge about the taxonomy and geographic distribution of most species, the so-called Linnean and Wallacean shortfalls, respectively (Hortal et al., 2015). Rigorous sampling and detailed information on species distributions are not available for the Macaronesian archipelagos, but Bayesian approaches such as that used by Gray and Cavers (2013) have the potential to explain the variation in meta-analyses where levels of taxonomic effort are unequal among islands.

The natural history of the Macaronesian Islands has attracted the attention of European naturalists, and noteworthy expeditions were made since the 17th century, including those by Hans Sloane, the founder of the British Museum, who made the earliest documented herbarium collections for the Madeira Island in 1687 (Menezes-de-Sequeira et al., 2010) and the explorations made by Captain James Cook who, during his first and second voyages, called at ports in Madeira, the Cabo Verde Islands, and the Azores (Francisco-Ortega et al., 2015). Several research projects focused on the history of plant exploration of the Macaronesia have been published during the last decade (Francisco-Ortega et al., 2008, 2010, 2015; Menezes-de-Sequeira et al., 2010; Santos-Guerra et al., 2011; Romeiras et al., 2014; Rico et al., 2017) and these have highlighted how the development of knowledge of the Cabo Verde flora lagged far behind the central Macaronesian archipelagos of the Canaries and Madeira for which knowledge of their endemic floras was far more developed by the mid-18th century (Romeiras et al., 2014).

The Cabo Verde Islands were uninhabited when the Portuguese colonized them in around 1462 (Albuquerque, 1991). Their generally dry climate was less favorable to human establishment compared to the other Macaronesian Islands, but, with the development of the transatlantic slave trade, the long-distance oceanic vessels needed ports of call, such as Cidade Velha (Santiago Island) and Mindelo (São Vicente Island) and the importance of this archipelago to Atlantic navigation increased considerably until the middle of the 19th century (Romeiras et al., 2011).

The first thorough botanical survey of the Cabo Verde archipelago was performed by the Portuguese naturalist João Silva Feijó who stayed in the islands from 1783 to 1797, and collected the earliest herbarium for this archipelago (Gardère et al., 2019). In the 19th century the islands began to attract more interest, and field expeditions were made by naturalists, including Chrétien Smith in 1816 (Tuckey, 1818); John Forbes in 1822 (Chevalier, 1935); Charles Darwin in 1832 (Fitzroy, 1839); Samuel Brunner in 1838 (Barbosa, 1961); Joseph Dalton Hooker in 1839 (Rico et al., 2017); and Theodor Vogel in 1841 (Webb, 1849). In the second half of the 19th century, other naturalists made important plant collections in the archipelago, including Carl August Bolle in 1851–52, returning to the archipelago in 1853, Friedrich Welwitsch when traveling to Angola in 1853 and upon his return in 1861, Richard Thomas Lowe in 1864 and 1866, and Johann Schmidt in 1851 (Lowe, 1868; Hiern, 1896). At the end of 19th century, João Cardoso stayed in the archipelago from 1883 until 1905 (Exell et al., 1952). Finally, in the 20th century, the Cabo Verde flora was the subject of several field expeditions, of which the most notable are Chevalier’s work in the first half of the century (1935) (Jacques-Félix, 1956) and, in 1956 and 1961, the work of Luís Grandvaux Barbosa (1914–1983), who made one of the most important collections of botanical material from the Cabo Verde Islands (Barbosa, 1961).

There can be a considerable lag between the collection and description of new taxa (Bebber et al., 2010) and collections differ markedly in their contribution to species discovery (Bebber et al., 2012). Despite the long-standing interest in the diversity and evolution of the Macaronesian flora and its acknowledged importance at both regional and global levels, there is no integrated study describing the historical process of collecting, discovery and description of its flora. Using as a case study the Cabo Verde endemic angiosperm flora, we apply a Bayesian approach to assess species discovery, through time and space within the nine islands of the archipelago.

Specifically, the present study aims to (i) document the description of the flora through time (ii) document the time to discovery of taxa (iii) identify the ‘big hitting collectors’ who have made the most significant contribution to species discovery in the archipelago, and (iv) understand how the characteristics of the archipelago (both natural and man-made) have shaped the discovery of new species in remote areas, such as oceanic islands. In particular, we test how the development of knowledge of the flora of Cabo Verde was influenced not only by the natural characteristics of the islands (e.g. area, age, maximum altitude and other topographical characteristics of the islands) but also by man-made/historical factors (e.g. the location of the two main harbors in Santiago and São Vicente).

## Materials and Methods

### Studied Area

The Cabo Verde archipelago encompasses the southernmost islands of Macaronesia, and is located c. 500 km west of Senegal (West Africa) and 1500 km south of the Canary Islands. This archipelago has ten main islands: Santo Antão, São Vicente, Santa Luzia (uninhabited) and São Nicolau constitute a northern group of islands; Santiago, Fogo, and Brava form a southern group; and Sal, Boavista, and Maio form an eastern group (Supplementary Figure S1). The northern and the southern islands are characterized by high mountains, namely Monte Gordo (1304 m) in São Nicolau; Pico da Antónia (1392 m) in Santiago; Tope de Coroa (1979 m) in Santo Antão; and Pico do Fogo (2829 m) in Fogo. Climatic factors related to altitude and exposure to the trade winds, are responsible for the different plant communities found in the archipelago (Romeiras et al., 2015).

### Studied Species

For this study, the list of Cabo Verde endemic angiosperm plants follows Romeiras et al. (2016), which considered 92 taxa (including infraspecific taxa) to be endemic to this archipelago. The only endemic pteridophyte species [*Dryopteris gorgonea* J.P. Roux (Dryopteridaceae)] was not included in our study. For the 91 endemic flowering plants, we used the year of collection for the earliest specimen reported, the year of collection of the type specimens (i.e. the specimens used for the original description of a taxa) and publication date as proxies for understanding species discovery in the Cabo Verde flora (Supplementary Table S1). This information was mainly obtained from JSTOR (2019) Plants^1^; the International Plant Names Index (2019)^2^; and the African Plant Database (2019)^3^ that represent the world’s largest databases of digitized plant specimens. Our database was complemented with analysis of floristic works for the Cabo Verde flora (e.g. Tuckey, 1818; Webb, 1849; Webb, 1849; Schmidt, 1852; Lobin, 1986; Chevalier, 1935; Pettersson, 1960; Brochmann et al., 1997) and an historic contextualization was carried out based on the “History of Cabo Verde” by Albuquerque (1991) and on recently studies by Romeiras et al. (2014) and Rico et al. (2017). The final dataset contains information on 91 Cabo Verde endemic vascular plants, including: where (island name) and when the type specimen was collected and by whom (collector), who described it and when, the date of the earliest collection, and its current distribution within the archipelago.

We generated and compared accumulation curves for the year of collection of (i) the earliest recorded specimen (ii) the type and (iii) the publication of the basionym for the endemic flora. We used these to determine the “description lag” – the time difference between date of collection of the earliest collection and date of publication of the protologue, following Bebber et al. (2012). We also identified key years for first records, for the collection of types and for the description of endemic diversity and we identified the individuals (the “big hitters” *sensu* Bebber et al., 2012), responsible for those advances. We also plotted the earliest record for endemics for each island in the archipelago.

### The Bayesian Approach

We used Bayesian methods to model the number of endemic species collected as a function of natural characteristics of the islands, as well as key man-made features. Based on historical information, we identified the harbors on the islands of Santiago and São Vicente as two of the features that most influenced the colonization of the islands and the patterns of collecting. As we were interested in identifying the natural and man-made features that could influence the development of collections, we excluded single island endemics (hereafter SIE) because their collection necessarily occurs in the islands where they are present and it is independent of the location of harbors. The proximity of an island to one of the major harbors may have led to an earlier identification of a SIE, but we were mainly concerned with how the attributes of islands (including those related to human activities) and their geographical arrangement (e.g., distance among islands) may have determined the way species were collected.

Given the characteristics of our dataset we could have analyzed the time series of the accumulation of endemic species identified per year in the nine islands. For some islands, however, this would have been a very short time series, and for two islands (Boavista and Maio), we would have had only one point since there is only one endemic species in each of these islands. Therefore, we adopted a different strategy that emphasizes the geography of the archipelago. We nevertheless consider the temporal component by analyzing the data for six time periods, starting in 1850 and ending in 1975 with time intervals of 25 years. These starting and ending years were chosen because the types of only a few species had been collected before 1850, and after 1975 the types of most species have already been collected (80% of species described).

For a given year, we modeled the number of endemic species as a function of (natural and artificial) characteristics of the islands where they were first recorded. Since we are dealing with a relatively small number of discrete observations, we assume for the likelihood a Poisson distribution. Accordingly, if the total number of species collected in island *i* at time *t* is *s*_*it*_, then *s*_*it*_∼Poisson(*λ*_*it*_); in order to avoid cluttering the equations with subscripts we omit *t* from now on, but it is implicit that each equation applies to one of the time periods considered. The parameter *λ_*i*_* is the outcome variable we want to model using a set of explanatory variables. As it usually done with generalized linear models (e.g. Gelman et al., 2013), we model λ*_*i*_* using a “log” link function

$$\text{log}\,\left(\lambda_{i}\right)=\gamma_{0}+\gamma_{1}\,x_{i\,1}+\gamma_{2}\,x_{i\,2}+\ldots +\gamma_{k}\,x_{i\,k},$$

where the *x*_*ij*_ are the explanatory variables to be considered for island *i* and the *γ_*j*_* the corresponding parameters to be estimated.

For the explanatory variables related to the natural characteristics of each island (see also the Discussion) we used: area, age, maximum altitude and the average value of the Terrain Ruggedness Index (hereafter TRI; Riley et al., 1999). The latter is a topographical characteristic of an island (the larger the value the more rugged an island is) that may determine its species richness by, for instance, creating a larger number of habitats. It may also reflect the difficulty in accessing different parts of the island thus, influencing the rate at which new species were collected.

As noted previously, we were also interested in historical features that could have conditioned the collection of species. One of these characteristics is the presence of a major harbor, a feature that determined the early colonization pattern of the archipelago, and which we modeled as an indicator variable that takes the value of one (1) for the island of Santiago and São Vicente, and the value of zero (0) for the other islands (i.e. those without a major harbor).

The number of endemic species in one island is likely to be correlated with the number of endemic species in the neighboring islands (Duarte et al., 2008) and, moreover, the geographical location of the islands is likely to play a major role in the way the collection of the species grew over time. In order to consider the spatial autocorrelation among the islands, we also developed models with a Gaussian Process (e.g., McElreath, 2015). The Gaussian Process is introduced in a model by considering a varying intercept (thus it becomes a hierarchical model), where each island has a different intercept *ψ*_*[i]*_ (the square brackets emphasize the hierarchical nature of this parameter in the model). The Gaussian process assumes that the *ψ*_*[i]*_ is given by a multivariate normal distribution with mean 0 and matrix of covariance Γ whose elements are

$${\text{ Γ }}_{i\,j}=\eta^{2}\,\exp\left(-\rho^{2}\,D_{i\,j}^{2}\right)+\delta_{i\,j}\,\sigma^{2},$$

where δ_*ij*_ is (the Kronecker delta function) equal to 1 when *i* = *j*, and 0 otherwise; this parameter also adds a covariance value when *i* = *j*, but because for the present problem each island contains only one observation, this extra term is in fact irrelevant and we consider it proportional to a constant (see also McElreath (2015), chapter 13). Thus, the final model is:

(1) $$s_{i}∼\text{Poisson}\,\left(\lambda_{i}\right),$$

(2) $$\text{log}\,\left(P_{i}\right)=\gamma_{0}+\gamma_{1}\,x_{i\,1}+\gamma_{2}\,x_{i\,2}+\ldots +\gamma_{k}\,x_{i\,k}+\psi_{\left[i\right]},$$

(3) ψ∼MVNormal⁢((0,…,0),
Γ
),

(4) $${\text{ Γ }}_{i\,j}=\eta^{2}\,\exp\left(-\rho^{2}\,D_{i\,j}^{2}\right)+0.01\,\delta_{i\,j},$$

(5) $$\gamma_{j}∼\text{Normal}\,\left(0,10\right),$$

(6) $$\eta^{2}∼\text{HalfCauchy}\,\left(0,1\right),$$

(7) $$\rho^{2}∼\text{HalfCauchy}\,\left(0,1\right),$$

where MVNormal stands for “multivariate normal distribution”. We assumed normal priors for *γ*_*j*_ and half Cauchy hyperpriors for η^2^ and *ρ*^2^. These priors were chosen so that their distributions are relatively flat in the region where the parameters are likely to have values different from zero, but also confined to a range of values that led to an efficient numerical sampling. We checked the choices for the priors and concluded that for a wide range of values they did not affect the results in a meaningful way.

The variables “altitude”, “age” and “TRI” are highly correlated (see Supplementary Figure S2 (linear scales) and Supplementary Figure S3 (logarithmic scales); in Supplementary Appendix I), therefore, we did not include them simultaneously in the same model. Instead, we constructed different models for each one and compared them using the WAIC (e.g. McElreath, 2015). The Gaussian process led to a considerable improvement according to the WAIC values, hence we included it in all models. Finally, we considered the importance of the presence of the harbors in Santiago and São Vicente islands. Thus, for the six time periods considered we compared the WAIC obtained with models with and without harbor. In summary, each of our models consisted of one of the variables “altitude,” “age,” “area” or “TRI,” plus the Gaussian process and either the presence or absence of a harbor, thus Eq. 2 above is of the form

$$\text{log}\,\left(P_{i}\right)=\gamma_{0}+\gamma_{1}\,x_{i\,1}+\gamma_{2}\,h\,a\,r\,b\,o\,u\,r+\psi_{\left[i\right]},$$

or

$$\text{log}\,\left(P_{i}\right)=\gamma_{0}+\gamma_{1}\,x_{i\,1}+\psi_{\left[i\right]},$$

where *x*_*i*_ is “altitude”, “age”, “area” or “TRI”. We denoted the models by using the acronyms “ag” (age), “al” (altitude), “ar” (area) and “tri” (TRI) followed, or not, by “h”, depending on whether the variable “*harbor*” was present. For instance, the model “*trih*” means that the variables “TRI” and *harbor* were used; the Gaussian process was present in all models thus we did not mention it explicitly in the models’ acronyms.

All the analyses were conducted in R version 3.5.1 (R Core Team, 2018) with the packages Rethinking (McElreath, 2015) and RStan (Stan Development Team, 2018).

## Results

The location and year of collection of the type specimen as well as of the earliest cited specimen, the collector of those specimens and the current distribution of each endemic taxon are summarized in Supplementary Table S1. A detailed list of the main collectors in the Cabo Verde Islands and their dates is provided in Supplementary Table S3.

**FIGURE 1:**
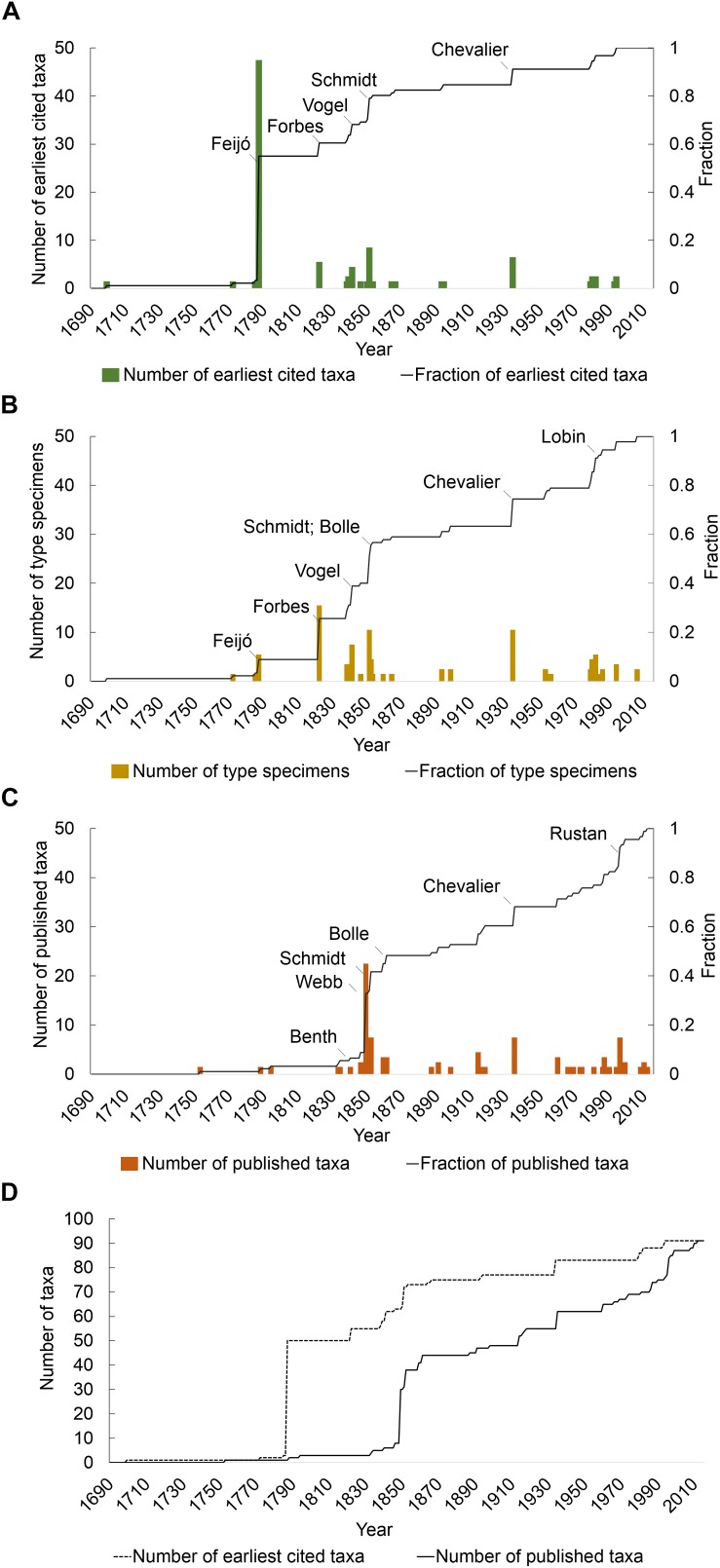
Discovery process of Cabo Verde vascular flora through time since 17th century, and the big hitting collectors. **(A)** Earliest cited material; **(B)** types per collector; **(C)** publication and description of new taxon; and **(D)** lag between date of earliest cited material (dotted line) and the description of the taxon (solid line). For the species that have more than one type specimen, only the first was represented.

Figure 1A illustrates the year of first collection for endemic taxa. João Feijó (1760–1824) was responsible for the first collection of more than 50% of the endemic flora in the 1780s. Johann Schmidt (1823–1905) was the second most significant contributor of first records of endemic taxa, collecting seven taxa in 1851. Other significant contributors responsible for the first collection of endemic taxa were John Forbes (1799–1823; five taxa in 1822); Johann Schmidt (1823–1905; seven taxa in 1851), Theodor Vogel (1812–1841; four taxa in 1841); and Auguste Chevalier (1873–1956; six taxa in 1934).

The date of collection of type specimens for endemic taxa (Figure 1B) followed a similar episodic pattern but with notable differences. Whilst Feijó, Forbes, Vogel, Schmidt and Chevalier were each also responsible for the collection of more than five type specimens, their relative importance differed when compared with the pattern for first records. Forbes and Vogel were the greatest contributors with 15 type specimens each, whilst Chevalier and Hooker with nine, Bolle with eight and Schmidt with seven types were also among the most significant contributors of type specimens. Wolfram Lobin (1951-) also collected six types between 1979 and 1982. Whilst Feijó was responsible for more first records than any other collector, only a small proportion of those were type specimens (six of the 48 species for which he was responsible for the first record have a specimen he collected as type).

With regards the description of endemic taxa in the Cabo Verde flora (Figure 1C), Philip Barker Webb (1793–1854) made the largest single contribution, his Niger flora (1849) responsible for 25 species or more than 27% of the endemic flora. Publications by Schmidt, Chevalier and Bolle each contributed more than five new taxa to the endemic flora (seven, six and six, respectively). More recently, new taxa were described by several authors with Lobin and Rustan contributing eight and six, respectively, new taxa, respectively.

A comparison of the cumulative curves for first records and description of endemic taxa reveals a significant lag between the two (Figure 1D). The average lag between first collection and description was 49 years. For 20% of taxa, there was a lag of more than 100 years between the first collection and description.

**FIGURE 2:**
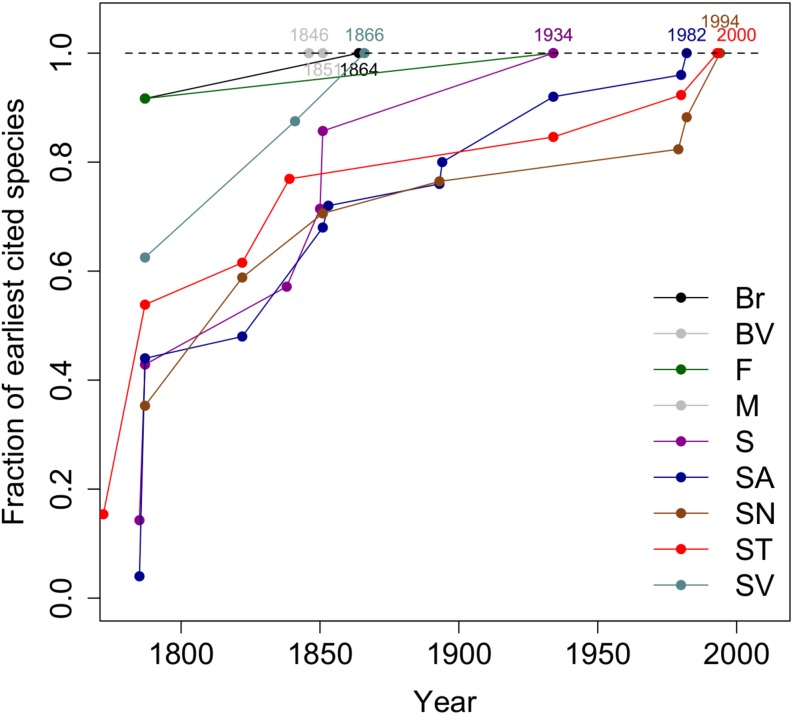
The temporal evolution of the fraction of the earliest cited material per island. Island abbreviations: Santo Antão (SA), São Vicente (SV), São Nicolau (SN) (Northern Group); Sal (S), Boavista (BV), Maio (M) (Eastern Group); Santiago (ST), Fogo (F), Brava (Br) (Southern Group).

The rate at which the endemic species have been collected on each of the nine islands since the 18th century is shown in Figure 2. For São Vicente all endemic species were collected by 1866. Boavista and Maio each have only one endemic species and in both cases, this was collected early on in the first half of the 19th century. Approximately 85% of endemic species were collected for the first time before the end of the 19th century.

We started our analysis of the impact of natural and artificial factors on the description of the endemic flora by comparing the performance of the models with and without harbor using the WAIC index (Table 1). For each of the first four periods analyzed, models with harbors included performed better, although both models including and excluding the presence of harbors had ΔWAIC less than 2 indicating substantial empirical support.

**TABLE 1:** Comparison of the WAIC values among the eight models for the six periods analyzed.

	**WAIC**	**ΔWAIC**	**Weight**
**1850**			
*tri.h*	*31.7*	*0*	*0.23*
*ar.h*	*31.8*	*0.2*	*0.21*
*al.h.*	*32.1*	*0.5*	*0.18*
*ag.h*	*32.2*	*0.5*	*0.17*
ag	33.7	2	0.08
ar	34	2.3	0.07
tri	34.4	2.7	0.06
al	34.4	2.7	0.05

**1875**			

*tri.h*	*40.7*	*0*	*0.22*
*ag.h*	*41.2*	*0.5*	*0.17*
*ar.h*	*41.3*	*0.6*	*0.17*
*al.h*	*41.8*	*1.1*	*0.13*
al	42.5	1.8	0.09
ag	42.6	1.9	0.09
tri	43	2.3	0.07
ar	43.3	2.6	0.06

**1900**			

*tri.h*	*40.6*	*0*	*0.21*
*al.h*	*41.1*	*0.5*	*0.17*
*ar.h*	*41.2*	*0.6*	*0.16*
*ag.h*	*41.4*	*0.8*	*0.14*
tri	42.3	1.7	0.09
al	42.4	1.8	0.09
ag	42.6	2	0.08
ar	42.7	2.1	0.07

**1925**			

*tri.h*	*40.6*	*0*	*0.21*
*al.h*	*41.1*	*0.5*	*0.16*
*ar.h*	*41.4*	*0.8*	*0.14*
*ag.h*	*41.4*	*0.8*	*0.14*
ag	42.1	1.5	0.1
al	42.3	1.7	0.09
tri	42.4	1.8	0.09
ar	42.6	2	0.08

**1950**			

*tri.h*	*42*	*0*	*0.18*
*al.h*	*42.1*	*0.1*	*0.17*
ag	42.1	0.1	0.17
*ag.h*	*42.5*	*0.5*	*0.14*
*ar.h*	*42.8*	*0.8*	*0.12*
tri	43.3	1.3	0.09
al	43.8	1.8	0.07
ar	44	2	0.07

**1975**			

*tri.h*	*43.4*	*0*	*0.2*
*ag.h*	*44*	*0.6*	*0.15*
*al.h*	*44.3*	*0.9*	*0.13*
ag	44.4	1	0.12
al	44.5	1.1	0.12
*ar.h*	*44.9*	*1.5*	*0.1*
tri	44.9	1.5	0.1
ar	45.1	1.7	0.09

*The names of the models denote the variables used according to the code: “ag” for age, “a” for altitude, “ar” for area, “tri” for TRI and “h” for presence of harbor. The results for the models including the variable “harbor” are in italic.*

Nevertheless, the ΔWAIC becomes progressively smaller for the most recent time periods. Thus, while the WAIC difference between the best model with “harbor” and the model without “harbor” in 1850 is ΔWAIC = 2, indicating considerably less evidence for the latter, for 1975 that difference is only ΔWAIC = 1. We interpret this decrease as a reduction in the importance of the presence of the harbors for discovery through time.

Among the several abiotic variables analyzed the results were similar for the models with all variables. We did not include all of them simultaneously in one single model because they were highly correlated (Supplementary Figure S2). Here we present results and concentrate our discussion only on the model including TRI (a measure of the island ruggedness) and the Gaussian process (to take into account spatial autocorrelation) because these were the models that performed consistently well for all periods.

Notice, however, that models including maximum altitude, age of the islands, their area, or simply the presence or absence of a major harbor always had a ΔWAIC smaller then 3, and typically smaller than 2, thus revealing considerable empirical evidence for these other models as well. Supplementary Appendix I provides further details on the implementation of the models and comparison of their results.

**FIGURE 3:**
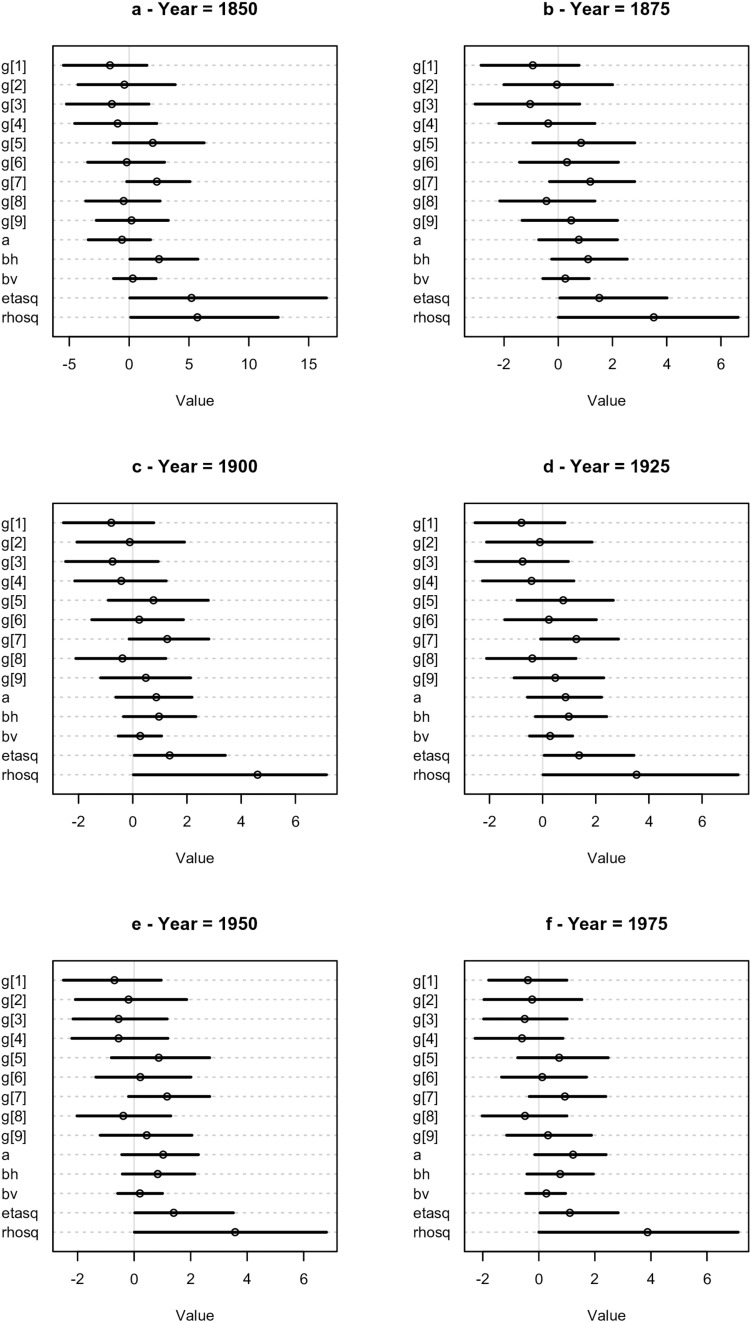
Dotchart plots of the posterior densities of the parameters of the tri. h model for the 6 periods analyzed. The “g”, “etasq” and “rhosq” correspond to the parameters of the Gaussian Process, “a” is the intercept, “bv” the term associated with the TRI variable and “bh” the term associated with the presence or absence of a major harbor.

Figure 3 shows the values of the parameters of the *trih* model for the 6 years analyzed. The posterior of the parameter associated with TRI, denoted “bv” in Figure 3, has both positive and negative values, but the mean is positive. This is not surprising given that this variable is correlated with the total number of species (Supplementary Appendix I). The posterior of “bh”, associated with the presence or absence of a major harbor, is mainly positive for the first period analyzed (1850). In subsequent periods its mean moves toward zero with a considerable part of the posterior distribution having negative values. The other parameters are related to the Gaussian Process, modeling the spatial autocorrelation as we now interpret.

**FIGURE 4:**
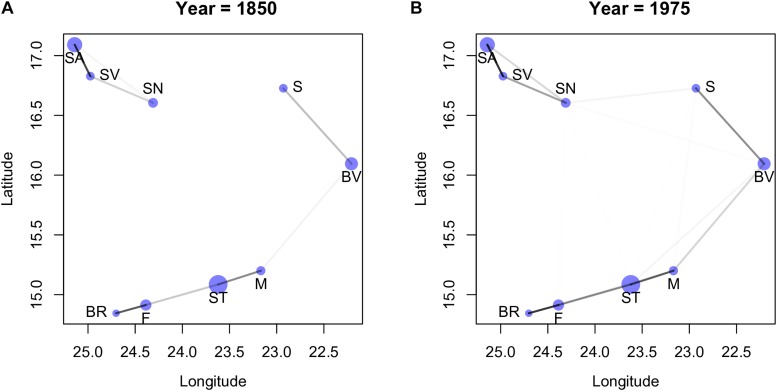
The dots correspond to the location of the islands in geographic space, and the size of the circles are proportional to the size of the islands. The darker the lines between the islands the larger the corresponding value in the correlation matrix (see main text). All pairs of islands have lines connecting them, however, in some cases the correlation values are so low that the lines are almost white, and thus very difficult to discern. Plot **(A)** was obtained with the values of 1850, and plot **(B)** with the values of 1975; the plots are very similar to all the other years. Island abbreviations: Santo Antão (SA), São Vicente (SV), São Nicolau (SN); Sal (S), Boavista (BV), Maio (M); Santiago (ST), Fogo (F), Brava (BR).

Using the posterior of the parameters *η*^2^ and *ρ*^2^ (denote in Figure 3 by “etasq” and “rhosq,” respectively), we estimated the covariance matrix, Γ (see Supplementary Figure S4 in Supplementary Appendix I where we show the values of the covariance obtained from the posterior). The correspondent correlation matrix, *R*, is easier to interpret (McElreath, 2015) and we consequently calculated the correlation matrices for all years, using the median values of the posteriors of *η*^2^ and *ρ*^2^ (we used the median mainly because *η*^2^ tends to have a rather skewed posterior). We observed that the values of the correlation matrices did not change considerably among the last 5 periods, thus we consider only the results for the first year, 1850, and last year, 1975 (see Supplementary Appendix I, for all years). Instead of showing the correlation matrices, we depict its values graphically in Figure 4 together with the geographical location of the islands (see Supplementary Figure S5 for all years). All island pairs are connected, but since the darkness of the line is proportional to the strength of the correlation, and because the correlations among some islands are very weak, the corresponding connecting lines are not (easily) visible. Visual inspection of the Figure 4 reveals two main groups of islands, one consisting of Santo Antão (SA), São Nicolau (SN) and São Vicente (SV), and the other consisting of the remaining islands. The correlations between the islands of São Nicolau and Sal, and Santiago and Maio and Boavista are stronger in 1975 than in 1850. The emergence of these specific clusters is not surprising given that they are formed by islands that are close together, but it is nevertheless notable that each cluster contains an island with a major harbor (Santiago and São Vicente).

**FIGURE 5:**
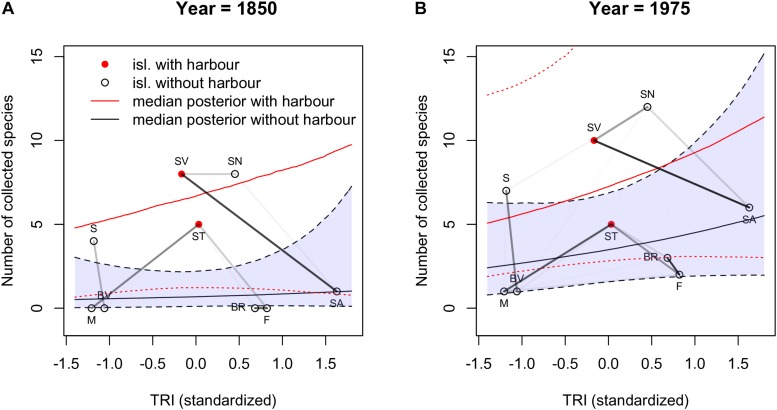
The number of collected species as a function of (standardized) TRI for 1850 and 1975, plots **(A)** and **(B)**. The values corresponding to islands with harbors (Santiago and São Vicente) are shown with red dots, the others are in black. The dotted line represents the mean of the average posterior predictive relationship between TRI and proportion of species collected. The dotted line corresponds to the median of the posterior for the islands with a harbor and the dashed line to the median of the posterior for islands without harbors. The gray shade denotes the 80% confidence intervals of the posterior of the islands with a major harbor and the blue shade the 80% confidence intervals of the posterior of the islands without a major harbor. To these results we superimposed the values of correlations of the spatial autocorrelation between the islands with darker lines corresponding to stronger correlations. Island abbreviations: Santo Antão (SA), São Vicente (SV), São Nicolau (SN); Sal (S), Boavista (BV), Maio (M); Santiago (ST), Fogo (F), Brava (BR).

To further explore the connection between these clusters and the proportion of endemic species sampled, we plotted the latter as a function of (standardized) TRI for 1850 and 1975 (Figure 5; see Supplementary Figure S6 for all years). In this figure the two red dots correspond to the values obtained for the islands with harbors (São Vicente and Santiago). We can interpret the cluster formed by São Vicente (SV), Santo Antão (SA) and São Nicolau (SN) as a group of islands that are close together and tend to have the number of collected species above the median value. The other cluster of islands show correlations that seem to be mostly determined by distance, but the strong correlation between Brava (BR) and Fogo (F) reflects islands with a fraction of collected species below the median.

## Discussion

The results of this paper highlight the episodic collection and description of the Cabo Verde endemic angiosperm flora and the importance of man-made features, notably harbors, in the accumulation of herbarium collections that underpinned the discovery of the Cabo Verde flora.

Until the late 19th century, the Cabo Verde archipelago was strategically important for victualing vessels from European countries (Romeiras et al., 2014). During this period the islands were visited by notable naturalists, including Smith (Tuckey, 1818), Forbes (Chevalier, 1935), Darwin (Darwin, 1936), Brunner (Barbosa, 1961), Hooker (Rico et al., 2017), Vogel (Webb, 1849) and Welwitsch (Hiern, 1896). However, their visits were short as they were all passing through on their way to botanically richer regions in Africa and South America. During the 19th century, few naturalists were focussed on investigating the flora of the Cabo Verde Islands. Two of those, namely Feijó at the end of the 18th century (Gardère et al., 2019) and Schmidt at the end of the 19th century (Exell et al., 1952) were, between them, responsible for the first records of more than 60% of the endemic flora (Figure 1A).

Whilst Feijó was a particularly significant figure in the discovery of the endemic flora, his specimens were largely overlooked as types for the description of new taxa. Indeed, of the almost 50 specimens collected by Feijó that constitute first records, only six were selected as type specimens. Feijó collected *Diplotaxis hirta, Echium vulcanorum, Periploca chevalieri, Tornabenea humilis* and *Verbascum cystolithicum* at the end of the 18th century, but none were described until the mid-20th century. This, in part, reflects the limited data and notably the lack of provenance data (i.e. location; island) associated with Feijó’s collections that limited their utility. Overall, there was a delay of over a century between first collection and description for 20% of endemic taxa. Taxon discovery is dependent on detailed, expert examination of herbarium material that often only occurs decades after its first collection (Bebber et al., 2010). The global analysis undertaken by Bebber et al. (2010) revealed an average discovery lag time of ca. 39 years; for the Cabo Verde flora, it is 49 years.

Bebber et al. (2012) investigated the contribution of individual collectors to plant species discovery, demonstrating that more than half of all type specimens were collected by less than 2 per cent of collectors. The disproportionate contribution of a few individuals to the discovery of the Cabo Verde flora is also apparent. Indeed, five of the 31 individuals who have collected in the archipelago (16%) accounted for 77% of first discoveries whilst six collectors (19%) account for 63% of type specimens. Bebber et al. (2012) found that this pattern persists through time. In Cabo Verde, we similarly find that “big hitting” collectors are not restricted in time to the 18th (i.e. Feijó) and 19th centuries (e.g. Forbes; Hooker; Vogel; Schmidt; Bolle: see Figure 1B) but are equally a phenomenon of contemporary botany. For example, in the late 20th century, Lobin, Rustan and Kilian made large plant collections in Cabo Verde Islands and contributed significantly to the collection of types of newly described species. Although the discovery of flowering plants in the Cabo Verde Islands has slowed, taxa continue to be discovered and a number of undescribed species are hypothesized to exist, particularly in habitats that are difficult to access, such as cliffs in the mountain areas where most of the islands’ endemics occur (Romeiras et al., 2015).

The species accumulation curves for individual islands (Figure 2) shows that prior to 1850, most species discoveries were sampled in Santiago, São Vicente and São Nicolau (an island in close proximity to São Vicente; Supplementary Figure S1). Portuguese settlers arrived at Santiago the largest island of the archipelago in 1462. Although São Vicente is a small island (7th largest in the archipelago) the harbor of the city of Mindelo (São Vicente) became an important commercial center during the 18th century. The port of Preguiça was one of the first settlements on São Nicolau, and it was established during the 18th century as a safe port between São Vicente and Sal Islands. These were the harbors where passing naturalists called and it was in the proximity of these harbors that their herbarium collections were made. Theodore Vogel is a case in point. Vogel was Chief Botanist to the “Niger Expedition” organized by the Horticultural Society of London. He arrived in the archipelago on June 3 of 1841 and visited São Vicente and Santo Antão between 6 to 18 of June (Hooker, 1849). When Vogel reached São Vicente Island, he was very disappointed and referred to the desolate aspect of that island noting that “after a search of 4 h, climbing several hills and crossing as many valleys, I only met with two plants *Tamarix senegalensis*, a shrub, and a low shrub-like Labiata (*Lavandula formosa*?)”. Later when Vogel explored Monte Verde, the only mountain in this island he found several species, making it “a pleasant spectacle, such as one would hardly have expected on an apparently desert island” (Webb, 1849). During his short visit, Vogel was responsible for four first records and for collecting 15 specimens that would subsequently be designated type specimens.

The discovery of the Cabo Verde flora was largely opportunistic, involving naturalists who took advantage of being in one or a few of the islands for a few days or weeks, and not through dedicated systematic exploration of the archipelago (Romeiras et al., 2014; Rico et al., 2017).

Such “opportunistic discovery” of the flora is likely to also explain the patterns of discovery for other archipelagos such as the Azores and the Canary Islands that were important for trade during the 18th and 19th centuries, for which there has been no thorough, systematic study of the entire flora, and for which new discoveries continue to be made.

In addition to the historical analysis, we also developed a Bayesian model that took into consideration the proportion of endemic species collected, the geological age and topographical characteristics of the islands, the geographic arrangement of the archipelago and the location of its main harbors. We considered several variables because the Cabo Verde archipelago has some peculiarities that set it apart from other archipelagos. According to the theory of Island Biogeography (MacArthur and Wilson, 1967), area and distance of an island to the mainland are major determinants of species richness. However, for the Cabo Verde archipelago, area does not relate to species diversity as predicted; instead the maximum altitude of an island is positively correlated with its species richness (Steinbauer et al., 2016) and maximum altitude is strongly negatively correlated with area (Duarte et al., 2008; Ramalho, 2011); see Supplementary Figure S2 and Supplementary Table S2. As Whittaker et al. (2008) have discussed, several characteristics of the islands determine their species richness. The distinctive pattern in the Cabo Verde archipelago may be due to the tropical dry climate, the steep elevational gradients and the north-east trade winds that are key factors in shaping species distributions (Romeiras et al., 2016).

We were unable to include climate variables such as precipitation or temperature in our analysis even though they are likely to be a major force determining the richness and distribution of the flora in Cabo Verde. There are several reasons for this. First, for some islands, climate data is limited; indeed most islands do not have weather stations. Second, our analysis require a time series that is not available and there is plenty evidence that climate has changed considerably in the last 200 years, to the point where present data would not provide precise information on how the weather was in the past two centuries in Cabo Verde (Monteiro et al., 2020). The use of information available in datasets such as WorldClim – Global Climate Data^4^ or CHELSA^5^, is typically at spatial scales that are not informative for the topography of the Cabo Verde Islands, wherein there is significant spatial variation with altitude and exposure to the trade winds being responsible for contrasting weather conditions over small distances.

The Bayesian analysis clearly supported the role of the main harbors in the initial stages in the development of knowledge of the Cabo Verde flora. Significantly, however, the importance of harbors diminished through time. We suggest that this reflects both improved transportation between islands, and the desire of collectors to more fully explore the archipelago once islands with easier access had been explored.

The Bayesian approach we adopt in this study provides a framework to analyze historical collections to better understand biases in the accumulation of herbarium collections and in the development of our contemporary understanding of biodiversity. With modifications, notably the selection of a set of variables appropriate to the system under investigation, it could be applied to other archipelagos. An important feature of the Bayesian model is that it takes into account the spatial autocorrelation among the islands which was accomplished by including a Gaussian process (McElreath, 2015). Inclusion of spatial autocorrelation is important since the proximity of an island to another with a major harbor may have facilitated the exploration of its flora. In addition, we can expect that islands that are close share conditions that determine their species richness and composition.

The model could also be used to investigate the discovery process in non-archipelago systems. For example, in West Africa, botanical collecting until the mid-18th century was restricted to the vicinity of trading posts located in coastal areas, where some form of colonial control and settlement was established (Romeiras et al., 2018). The discovery of the flora in this region could be modeled through substituting the “presence of a major harbor” with one relating to proximity to the trading posts; depending on data availability this variable could be continuous or categorical.

In this paper, we have applied statistical procedures to address an eminently human historical question concerning the development of herbarium collections and biodiversity knowledge during the colonization of an archipelago. The results presented here demonstrate that the discovery of new species is determined by natural factors but also by historical contingencies. The Cabo Verde archipelago provides an example where recent human colonization led to the development of plant collections that were shaped by the development of the archipelago’s human infrastructure, notably the location of the harbors and the establishment of the first settlements. Understanding the historical process of species discovery and the biases inherent in that, can help to shape our future efforts to document the flora of archipelagos such as the Cabo Verde Islands that are characterized by a rich but vulnerable endemic flora.

## Data Availability Statement

All datasets generated for this study are included in the article/Supplementary Material.

## Author Contributions

MR, MC, and LBÁ conceptualized the study, designed the methodology, and analyzed the results. MR, MD, and SC prepared the Cabo Verde dataset. FD and LBÁ contributed to the Bayesian analysis. MR and LBÁ wrote and prepared the draft. MR, MC, MD, and LBÁ wrote, reviewed, and edited the manuscript. All authors have reviewed and approved the submitted version of this manuscript.

## Conflict of Interest

The authors declare that the research was conducted in the absence of any commercial or financial relationships that could be construed as a potential conflict of interest.

## Footnotes

1 https://plants.jstor.org/collection/TYPSPE

2 https://www.ipni.org/

3 http://www.ville-ge.ch/musinfo/bd/cjb/africa/recherche.php?langue=an

4 http://worldclim.org/

5 http://chelsa-climate.org/
