# Supplementary material for: Bayesian Methods to Analyze Historical Collections in Time and Space: A Case Study Using Cabo Verde Endemic Flora
Source: Front Plant Sci. 2020 Mar 13;11:278. doi: 10.3389/fpls.2020.00278 (PMC7083154; doi:10.3389/fpls.2020.00278)
Supplement: Supplementary file 1 [file Data_Sheet_1.pdf]

Bayesian methods to analyse historical collections in time and space: a case study using Cabo Verde endemic flora

Running title: Bayesian methods to analyse historical collections

Maria M. Romeiras[1,2*], Mark Carine[3], Maria Cristina Duarte[,2], Silvia Catarino[1], Filipe S. Dias[4,5] and Luís Borda-de-Água[4,5]

Supplementary Material: captions

Table S1. Information on type specimens of the Cabo Verde endemic plants and the actual distribution of each taxon in the archipelago.

Table S2. Natural characteristics of the islands. TRI stands for Terrain Ruggedness Index, a measure of the ruggedness of an island (see main text for details) and SIE for "Single island endemics".

Table S3. List of the main collectors and periods they stayed in Cabo Verde Islands, until the first half of the 20th century.

Figure S1. The Cabo Verde Archipelago.

Figure S2. The relationship between the linear values of age of the islands, their maximum altitude, area, TRI and the presently known number of endemic species. All values are in arbitrary unities.

Figures S3. The relationship between the logarithmic values of age of the islands, their maximum altitude, area, TRI and the presently known number of endemic species. All values are in arbitrary unities.

Figure S4. One hundred sampled values of the covariance sampled from the posterior of η^2 and ρ^2, for several values of distance (grey lines), for the six years studied. The black line corresponds to the median value of 10,000 samples.

Figure S5. The dots correspond to the location of the islands in geographic space, and the size of the circles are proportional to the size of the islands. The darker the lines between the islands the larger the corresponding value in the correlation matrix (see main text). All pairs of islands have lines connecting them, however, in some cases the correlation values are so low that the lines are almost white, and thus very difficult to discern. Notice that the plots are very similar to all years. Island abbreviations: Santo Antão (SA), São Vicente (SV), São Nicolau (SN); Sal (S), Boavista (BV), Maio (M); Santiago (ST), Fogo (F), Brava (BR).

Figure S6. The number of collected species as a function of (standardized) TRI for all time periods, plots a-f. The values corresponding to islands with harbours (Santiago and São Vicente) are shown with red dots, the other are in black. The dotted line represents the mean of the average posterior predictive relationship between TRI and proportion of species collected. The dot line corresponds to the median of the posterior for the islands with harbour and the dashed line to the median of the posterior for islands without harbour. The grey shade denotes the 80% confidence intervals of the posterior of the islands with a major harbour and the blue shade the 80% confidence intervals of the posterior of the islands without a major harbour; where these two shaded regions intercept there is a darker shaded region. To these results we superimposed the values of correlations of the spatial autocorrelation between the islands with darker lines corresponding to stronger correlations. Island abbreviations: Santo Antão (SA), São Vicente (SV), São Nicolau (SN); Sal (S), Boavista (BV), Maio (M); Santiago (ST), Fogo (F), Brava (BR).

Table S1. Information on type specimens of the Cabo Verde endemic plants and the actual distribution of each taxon in the archipelago.

Family / Scientific name	Protologue (publication and basionym where applicable)	Type Specimen(s) (1)			Earliest cited material (if different from type specimens) (1)			Species distribution (2)
		Island (2)	Date	Collector (3)	Island (2)	Date	Collector (3)	
**Apiaceae**								
Tornabenea annua Bég.	Ann. Mus. Civico Storia Nat. Giacomo Doria ser. 3, 8: 39 (1918).	ST	1898	Fea	ST	1839	Hooker	ST
Tornabenea bischoffii J.A.Schmidt	Beitr. Fl. Cap Verd. Ins.: 254 (1852).	SA	1851	J.A.Schmidt	-	-	-	SA
Tornabenea humilis Lobin & K.H.Schmidt	Sommerfeltia 24: 83 (1997).	F	1982	Lobin	F	1783-1789	Feijó*	F
Tornabenea insularis (Parl.) Parl.	Hooker's J. Bot. Kew Gard. Misc. 2: 370 (1850). Bas.: *Tetrapleura insularis* Parl., Niger Fl. [W. J. Hooker]. 131 (1849).	SV	1841	Vogel	Br	1783	Feijó	SV, SN, BR
Tornabenea ribeirensis K.H.Schmidt & Lobin	Feddes Repert. 110(1-2): 8 (1999).	SN	1982	Lobin	N	1979	Lobin	SN
Tornabenea tenuissima (A.Chev.) A.Hansen & Sunding	Fl. Macaronesia, ed. 2, 1: 92 (1979). Bas.: *Melanoselinum tenuissimum* A.Chev., Bull. Mus. Natl. Hist. Nat. 1935, Ser. II. vii. 143.	F	1934	Chevalier	-	-	-	F
**Apocynaceae**								
Cynanchum daltonii (Decne.) Liede & Meve	Kew Bull. 67(4): 752 (2012). Bas.: *Sarcostemma daltonii* Decne., Niger Fl. [W. J. Hooker]. 149. t. 14. (1849).	ST; SA, SV	1822, 1839; 1841	Forbes, Hooker; Vogel	Br; F	1783; 1783-84, 1786	Feijó	SA, SV, SN, BV, ST, F, BR
Periploca chevalieri Browicz	Arb. Kornick., Polon. xi. 38 (1966).	F	1934	Chevalier	Br; F	1783; 1783-84, 1786	Feijó*	SA, SN, ST, F, BR
**Arecaceae**								
Phoenix atlantica A.Chev. [a]	Bull. Mus. Natl. Hist. Nat. 1935, Ser. II. vii. 137.	S, ST	1934	Chevalier	-	-	-	S, BV, M, ST
**Asparagaceae**								
Asparagus squarrosus J.A.Schmidt	Beitr. Fl. Cap Verd. Ins. 165 (1852).	SA	1851	J.A.Schmidt	ST	1839	Hooker	SA, SV, SN, S, BV, M
Dracaena draco (L.) L. subsp. c*aboverdeana* Marrero Rodr. & R.S. Almeida	Int. J. Geobot. Res. 2: 36 (2012).	SA	2006	A.Marrero, R.Almeida & J. Caujapé	SA	1788-89	Feijó	SA, SV, SN, ST, F, BR

**Asteraceae**								
Artemisia gorgonum Webb	Niger Fl. [W. J. Hooker]. 142. (1849).	F	1783-84, 1786	Feijó*	-	-	-	SA, ST, F,
Asteriscus daltonii (Webb) Walp. subsp. *vogelii* (Webb) Greuter	Fl. Medit. 7: 46 (1997). Bas.: *Odontospermum vogelii* Webb, Niger Fl. [W. J. Hooker]. 140. (1849).	SV	1822; 1841	Forbes; Vogel	Br; F; SA	1783; 1783-84, 1786; 1785	Feijó*	SA, SV, SN, M, ST, F, BR
Asteriscus daltonii (Webb) Walp. subsp. *daltonii*	Ann. Bot. Syst. (Walpers) 2(5): 844 (1852). Bas.: *Odontospermum daltonii* Webb, Niger Fl. [W. J. Hooker]. 140. (1849).	SA, ST	1822; 1839	Forbes; Hooker	-	-	-	ST
Asteriscus smithii (Webb) Walp.	Ann. Bot. Syst. (Walpers) 2(5): 844 (1852). Bas.: *Odontospermum smithii* Webb, Niger Fl. [W. J. Hooker]. 139. (1849).	SN	1822	Forbes	-	-	-	SN
Conyza feae (Beg.) Wild	Bol. Soc. Brot. sér. 2, 43: 256 (1969). Bas.: *Nidorella feae* Bég. Ann. Mus. Nat. Hist. Genova, ser. 3 8: 50. 1917.	F	1898	Fea	Br; F	1784; 1783-84	Feijó*	SA, SV, SN, ST, F, BR
Conyza pannosa Webb	Niger Fl. [W. J. Hooker]. 135. (1849).	SV	1841	Vogel	?	1783-1789	Feijó*	SA, SV, SN, ST, BR
Conyza schlechtendalii Bolle	Bonplandia 7: 294 (1859).	SN	1851	Bolle	-	-	-	SN
Conyza varia (Webb) Wild	Bol. Soc. Brot. sér. 2, 43: 255 (1969). Bas.: *Erigeron varius* Webb, Niger Fl. [W. J. Hooker]. 134. (1849).	SN	1822	Forbes	-	-	-	SA, SV, SN, F, BR
Helichrysum nicolai N.Kilian, Galbany & Oberpr.	Folia Geobot. 45(2): 188 (183-199) (2010).	SN	1994	N.Kilian & Leyens	-	-	-	SN
Launaea gorgadensis (Bolle) N.Kilian	Willdenowia 18(1): 167 (1988). Bas.: *Sonchus gorgadensis* Bolle, Bonplandia 7: 298 (1859).	SN, SA	1851; 1852-53	Bolle	-	-	-	SA, SV, SN
Launaea picridioides (Webb) B.L.Rob.	Proc. Amer. Acad. Arts 49: 517 (1913). Bas.: *Rhabdotheca picridioides* Webb, Niger Fl. [W. J. Hooker]. 146. (1849).	SN; SV	1822; 1841	Forbes; Vogel	?	1783-1789	Feijó*	SA, SV, SN
Launaea thalassica N.Kilian, Brochmann & Rustan	Willdenowia 16(2): 491 (1987).	Br	1986	N.Kilian	Br	1864	Lowe	BR
Phagnalon melanoleucum Webb	Niger Fl. [W. J. Hooker]. 135. t. 9. (1849).	SV	1841	Vogel	-	-	-	SA, SV, SN, ST, F
Pulicaria burchardii Hutch. subsp. *longifolia* E.Gamal-Eldin	Phanerogam. Monogr., 14: 283 (1981).	S	1934	Dinklage	S	1850; 1851-52	J.A.Schmidt; Bolle	S

Pulicaria diffusa (Shuttlew. ex S.Brunn.) Pett.	Commentat. Biol. 22(9) (Coll. Vasc. Pl. C. Verde Isl.): 58 (1960). Bas.: *Francoeuria diffusa* Shuttlew. ex Brunn., Flora 23(1, Beibl.): 72 (1840).	S	1838	Brunner	?	1783-1789	Feijó*	S, BV, M, ST, F
Sonchus daltonii Webb	Niger Fl. [W. J. Hooker]. 144. t. 15. (1849).	ST, SV	1839; 1841	Hooker; Vogel	?	1783-1789	Feijó*	SA, SV, SN, ST, F
Tolpis farinulosa (Webb) Walp.	Ann. Bot. Syst. (Walpers) 2(5): 955 (1852). Bas.: *Schmidtia farinulosa* Webb, Niger Fl. [W. J. Hooker]. 143. (1849).	SV	1841	Vogel	F	1783-84, 1786	Feijó*	SA, SV, ST, F, BR
**Boraginaceae**								
Echium hypertropicum Webb	Niger Fl. [W. J. Hooker]. 155. (1849).	?	1783-1789	Feijó*	-	-	-	ST, BR
Echium stenosiphon Webb subsp. *glabrescens* (Pett.) Romeiras & Maria C.Duarte	Taxon 60(5): 1383 (2011). Bas.: *Echium glabrescens* Pett., Commentat. Biol. xxii. No. 9 (Coll. Vasc. Pl. C. Verde Isl.) 39 (1960).	SN	1953	H.Lindberg	N	1822	Forbes	SN
Echium stenosiphon Webb subsp. *lindbergii* (Pett.) Bramwell	Lagascalia 2(1): 97 (1972). Bas.: *Echium lindbergii* Pett., Commentat. Biol. xxii. No. 9 (Coll. Vasc. Pl. C. Verde Isl.) 36 (1960).	SA	1953	H.Lindberg	?	1783-1789	Feijó*	SA
Echium stenosiphon Webb subsp. *stenosiphon*	Niger Fl. [W. J. Hooker]. 155. t. 15. (1849).	SV	1841	Vogel	?	1783-1789	Feijó*	SV
Echium vulcanorum A.Chev.	Rev. Bot. Appl. Agric. Trop. 15: 915 (1935).	F	1934	Chevalier	F	1786	Feijó	F
**Brassicaceae**								
Diplotaxis antoniensis Rustan	Nordic J. Bot. 16(1): 35 (1996).	SA	1980	Rustan	-	-	-	SA
Diplotaxis glauca (J.A.Schmidt) O.E.Schulz	Bot. Jahrb. Syst. 54(3, Beibl. 119): 55 (1916). Bas.: *Sinapidendron glaucum* J.A.Schmidt, Beitr. Fl. Cap Verd. Ins. 267 (1852).	BV	1851	J.A.Schmidt	-	-	-	S, BV
Diplotaxis gorgadensis Rustan subsp. *brochmannii* Rustan	Nordic J. Bot. 16(1): 41 (1996).	SA	1980	Rustan	SA	1934	Chevalier	SA
Diplotaxis gorgadensis Rustan subsp. *gorgadensis*	Nordic J. Bot. 16(1): 38 (1996).	SA	1982	Rustan & Brochmann	-	-	-	SA
Diplotaxis gracilis (Webb) O.E.Schulz	Bot. Jahrb. Syst. 54(3, Beibl. 119): 56 (1916). Bas.: *Sinapidendron gracile* Webb, Niger Fl. [W. J. Hooker]. 99. t. 1. (1849).	SN	1822	Forbes	?	1783-1789	Feijó*	SN

Diplotaxis hirta (A.Chev.) Rustan & L.Borgen	Bocagiana 47: 4 (1979). Bas.: *Sinapidendron hirtum* A.Chev., Bull. Mus. Natl. Hist. Nat. 1935, Ser. II. vii. 141.	F	1934	Chevalier	F	1783-84	Feijó*	F
Diplotaxis sundingii Rustan	Nordic J. Bot. 16(1): 47 (1996).	SN	1982	Rustan & Brochmann	-	-	-	SN
Diplotaxis varia Rustan	Nordic J. Bot. 16(1): 47 (1996).	ST	1980	Rustan	-	-	-	ST, BR
Diplotaxis vogelii (Webb) Cout.	Herb. Gorg. Cat. i. 284 (1914). Bas.: *Sinapidendron vogelii* Webb, Niger Fl. [W. J. Hooker]. 100. t. 2. (1849).	SV	1841	Vogel	-	-	-	SV
Erysimum caboverdeanum (A.Chev.) Sunding	Garcia de Orta, Ser. Bot. 2(1): 11 (1974). Bas.: *Matthiola caboverdeana* A.Chev., Bull. Mus. Natl. Hist. Nat. 1935, Ser. II. vii. 139.	F	1934	Chevalier	-	-	-	F
Lobularia canariensis (DC.) L.Borgen subsp. *fruticosa* (Webb) L.Borgen	Opera Bot. 91: 70 (1987). Bas.: *Koniga fruticosa* Webb ex Christ, Bot. Jahrb. Syst. 9(1): 93 (1887).	SN	1851	Bolle	SA; F	1785; 1787	Feijó*	SA, SN, ST, F, BR
Lobularia canariensis (DC.) L.Borgen subsp. *spathulata* (J.A.Schmidt) L.Borgen	Opera Bot. 91: 80 (1987). Bas.: *Koniga spathulata* J.A.Schmidt, Beitr. Fl. Cap Verd. Ins. 266 (1852).	SV	1851	J.A.Schmidt	?	1783-1789	Feijó*	SV, SN
**Campanulaceae**								
Campanula bravensis (Bolle) A.Chev.	Rev. Bot. Appl. Agric. Trop. 15: 889 (1935). Bas.: *Campanula jacobaea* var. *bravensis* Bolle, Bonplandia 9: 51. 1861.	Br	1852	Bolle	F	1786	Feijó*	ST, F, BR
Campanula jacobaea C.Sm. ex Webb	Niger Fl. 148. t. 12. (1849)	SN, SA; ST; SV	1822; 1832, 1839; 1841	Forbes; Darwin, Hooker; Vogel	?	1788	Feijó*	SA, SV, SN, ST
**Caryophyllaceae**								
Paronychia illecebroides Webb	Niger Fl. [W. J. Hooker]. 106. t. 7. (1849).	SA; ST; SV	1822; 1839; 1841	Forbes; Hooker; Vogel	SA	1785	Feijó*	SA, SV, SN, BV, M, ST, F
Polycarpaea gayi Webb	Niger Fl. [W. J. Hooker]. 104. (1849).	SA; S	1785; 1838-1840	Feijó*; Brunner	-	-	-	SA, SV, SN, S, ST, F
**Cistaceae**								
Helianthemum gorgoneum Webb	Niger Fl. [W. J. Hooker]. 102. (1849).	Br; F	1784; 1783-84, 1786	Feijó*	-	-	-	SA, F, BR

**Crassulaceae**								
Aeonium gorgoneum J.A.Schmidt	Beitr. Fl. Cap Verd. Ins. 258 (1852).	SA	1851	J.A.Schmidt	-	-	-	SA, SV, SN
Umbilicus schmidtii Bolle	Bonplandia 7: 245 (1859).	SA, SN	1852	Bolle	F	1783-84	Feijó*	SA, SN, ST, F
**Cyperaceae**								
Carex antoniensis A.Chev.	Rev. Bot. Appl. Agric. Trop. 15: 1032 (1935).	SA	1934	Chevalier	-	-	-	SA
Carex paniculata L. subsp. *hansenii* Lewej. & Lobin	Senckenberg. Biol. 67(4–6): 440 (1987).	SA	1984	Heintze	SA	1934	Chevalier	SA
**Euphorbiaceae**								
Euphorbia tuckeyana Steud. ex Webb	Niger Fl. [W. J. Hooker]. 177 (1849).	ST; SV; F	1783-84; 1839; 1841	Feijó; Hooker; Vogel	-	-	-	SA, SV, SN, S, BV, ST, F, BR
**Fabaceae**								
Lotus alianus J.H.Kirkbr.	Pakistan J. Bot. 42(Sp. Issue): 1 (-5) (2010).	SA	1956	L.A.Grandvaux Barbosa	SV	1866	Lowe	SA, SV
Lotus arborescens Lowe ex Cout.	Herb. Gorg. Cat. i. 289 (1914).	SN	1864	Lowe	?	1783-1789	Feijó*	SN
Lotus brunneri Webb	Niger Fl. [W. J. Hooker]. 119. t. 3. (1849).	S	1838	Brunner	-	-	-	SV, S, BV, M
Lotus jacobaeus L.	Sp. Pl. 2: 775 (1753).	ST	1699	Willem Adriaan van der Stel (seeds)	-	-	-	ST, F
Lotus latifolius Brand	Bot. Jahrb. Syst. 25(1-2): 202 (1898).	SA	1853	Bolle	-	-	-	SA
Lotus purpureus Webb	Niger Fl. [W. J. Hooker]. 118. t. 6. (1849).	SN	1822	Forbes	F	1783-84, 1786	Feijó*	SA, SV, SN, BV, ST, F, BR
Teline stenopetala (Webb & Berthel.) Webb & Berthel. subsp. *santoantaoi* Marrero Rodr.	Bot. Macaronés. 27: 90 (-98) (2008).	SA	2006	A.Marrero	SA	1851	J.A.Schmidt	SA
**Frankeniaceae**								
Frankenia ericifolia C.Sm. ex DC. subsp. *caboverdeana* Brochmann, Lobin & Sunding	Nordic J. Bot. 15(6): 620 (1996).	SN	1979	Lobin	SA	1851	J.A.Schmidt	SA, SV, SN
Frankenia ericifolia C.Sm. ex DC. subsp. *montana* Brochmann, Lobin & Sunding	Nordic J. Bot. 15(6): 622 (1996).	SN	1986	N.Kilian	N	1822	Forbes	SN

**Gentianaceae**								
Centaurium tenuiflorum (Hoffmanns. & Link) Fritsch subsp. *viridense* (Bolle) O.Erikss., A.Hansen & Sunding	Fl. Macaronesia, ed. 2 1: 92 (1979). Bas.: *Erythraea viridensis* Bolle, Bonplandia 9: 52 (1861).	Br	1852	Bolle	?	1783-1789	Feijó*	ST, F, BR
**Lamiaceae**								
Lavandula rotundifolia Benth.	Labiat. Gen. Spec. 150 (1833).	SN	1822	Forbes	F; SA	1783-84, 1786; 1786	Feijó*	SA, SV, SN, ST, F
Micromeria forbesii Benth.	Labiat. Gen. Spec. 376 (1834).	SN	1822	Forbes	Br; F	1784; 1783-84, 1786	Feijó*	SA, SN, ST, F, BR
**Papaveraceae**								
Papaver gorgoneum Cout. subsp. *gorgoneum* [b]	Herb. Gorg. Cat. i. 284 (1914).	SN	1893	Cardoso	?	1783-1789	Feijó*	SN, F
Papaver gorgoneum Cout. subsp. *theresias* Kadereit & Lobin	Nordic J. Bot. 9(6): 646 (1990).	SA	1980	Lobin	?	1783-1789	Feijó	SA
**Plantaginaceae**								
Campylanthus glaber Benth. subsp. *glaber*	Prodr. [A. P. de Candolle] 10: 508 (1846).	SN; ST	1822; 1839	Forbes; Hooker	Br; F	1784; 1786	Feijó*	SA, SV, SN, ST, F, BR
Campylanthus glaber Benth. subsp. *spathulatus* (A.Chev.) Brochmann, N.Kilian, Lobin & Rustan	Sommerfeltia 24: 277 (1997). Bas.: *Campylanthus spathulatus* A.Chev., Rev. Bot. Appl. Agric. Trop. 1935, xv. 897.	SA	1934	Chevalier	SA	1894	Cardoso	SA
Globularia amygdalifolia Webb	Niger Fl. [W. J. Hooker]. 133. (1849).	?	1783-1789	Feijó*	-	-	-	SA, SN, ST, F, BR
Kickxia elegans (G.Forst.) D.A.Sutton subsp. *dichondrifolia* (Benth.) Rustan & Brochmann	Sommerfeltia 24: 284 (1997). Bas.: *Linaria dichondrifolia* Benth., Prodr. [A. P. de Candolle] 10: 270 (1846).	SN	1822	Forbes	?	1783-1789	Feijó*	SA, SV, SN, ST
Kickxia elegans (G.Forst.) D.A.Sutton subsp. *elegans*	Revis. tribe Antirrhineae: 211 (1988). Bas.: *Antirrhinum elegans* G. Forst., De Plantis Magellanicis et Atlanticis Commentationes: 50, no. 97. Mar-Apr 1788.	ST	1772	Forster	ST	1772	Forster	SA, SV, SN, S, BV, M, ST, F, BR
Kickxia elegans (G.Forst.) D.A.Sutton subsp. *webbiana* (Sunding) Rustan & Brochmann	Sommerfeltia 24: 286 (1997). Bas.: *Kickxia webbiana* Sunding, Garcia de Orta, Ser. Bot. 2(1): 21 (1974).	SA	1851	J.A.Schmidt	?	1783-1789	Feijó*	SA

**Plumbaginaceae**								
Limonium braunii (Bolle) A.Chev.	Rev. Bot. Appl. Agric. Trop. 15: 928 (1935) Bas.: *Statice braunii* Bolle, Index Seminum [Berlin] 4 (1861).	SA	1852	Bolle	?	1783-1789	Feijó*	SA, SN, F, BR
Limonium brunneri (Webb) Kuntze	Revis. Gen. Pl. 2: 395 (1891). Bas.: *Statice brunneri* Webb, Niger Fl. [W. J. Hooker]. 170. (1849).	S	1838	Brunner	Br; F; SA	1784; 1783-84, 1786; 1785	Feijó*	SV, S
Limonium jovibarba (Webb) Kuntze	Revis. Gen. Pl. 2: 395 (1891). Bas.: *Statice jovibarba* Webb ex Boiss., Prodr. [A. P. de Candolle] 12: 665 (1848).	SV	1841	Vogel	-	-	-	SV, SN
Limonium lobinii N.Kilian & Leyens	Willdenowia 24(1–2): 59 (1994).	ST	1994	Heckel	ST	1993	Duarte	ST
Limonium sundingii Leyens, Lobin, N.Kilian & Erben	Willdenowia 25(1): 208 (1995).	SN	1994	N.Kilian & Leyens	-	-	-	SN
**Poaceae**								
Aristida cardosoi Cout.	Herb. Gorg. Cat. i. 273 (1914).	SN, SA	1893	Cardoso	-	-	-	SA, SV, SN, S, BV, M, ST, F, BR
Brachiaria lata (Schumach.) C.E.Hubb. subsp. *caboverdeana* Conert & C.Köhler	Senckenberg. Biol. 67(4–6): 432 (1987).	ST	1979	Lobin	?	1783-1789	Feijó*	SV, SN, BV, ST
Eragrostis conertii Lobin	Willdenowia 16(1): 143 (1986).	SA	1982	Lobin	SV	1841	Vogel	SA, SV, SN, ST, F
Sporobolus minutus Link subsp. *confertus* (J.A.Schmidt) Lobin, N.Kilian & Leyens	Willdenowia 25(1): 192 (1995). Bas.: *Sporobolus confertus* J.A.Schmidt, Beitr. Fl. Cap Verd. Ins. 142 (1852).	S	1851	J.A.Schmidt	-	-	-	S, M
**Sapotaceae**								
Sideroxylon marginatum (Decne. ex Webb) Cout.	Cat. Herb. Gorg. Suppl. ii. 43 (1915). Bas.: *Sapota marginata* Decne. ex Webb, Niger Fl. [W. J. Hooker]. 169. t. 13. (1849).	ST	1839	Hooker	F	1786	Feijó	SA, SV, SN, S, BV, ST, F, BR
**Scrophulariaceae**								
Verbascum capitis-viridis Hub.-Mor.	Bauhinia 5(1): 11 (1973).	SV	1859-1860	Wawra & Maly	?	1788	Feijó*	SA, SV, SN, BV, M, ST
Verbascum cystolithicum (Pett.) Hub.-Mor.	Bauhinia 5(1): 12 (1973). Bas.: *Celsia cystolithica* Pett., Commentat. Biol. xxii. No. 9 (Coll. Vasc. Pl. C. Verde Isl.) 42 (1960).	F	1954	H.Lindberg	F	1786	Feijó	F

**Solanaceae**								
Solanum rigidum Lam.	Tabl. Encycl. ii. 23 (1794).	?	?	?	F	1783-84, 1786	Feijó	SA, SV, SN, BV, M, ST, F, BR
Withania chevalieri A.E.Gonç.	Garcia de Orta, Ser. Bot. 14(1): 149 (1999).	S	1934	Chevalier	?	1783-1789	Feijó	SA, SV, S, F
**Urticaceae**								
Forsskaolea procridifolia Webb	Niger Fl. [W. J. Hooker]. i. 179. (1849).	SN; ST; SA, SV	1822; 1839; 1841	Forbes; Hooker; Vogel	Br; F	1784, 1783-84, 1786	Feijó*	SA, SV, SN, S, M, ST, F, BR
**Zygophyllaceae**								
Fagonia mayana Schlecht.	Bot. Zeitung (Berlin) 9: 844 (1851).	M	1846	C.Pabst	-	-	-	BV, M

Notes.

(1) Islands, dates and collectors are separated by semicolons when corresponding to different collection events. Early's collections were usually classified under a different name.

(2) Island abbreviations: Santo Antão (SA), São Vicente (SV), São Nicolau (SN) (Northern Group); Sal (S), Boavista (BV), Maio (M) (Eastern Group); Santiago (ST), Fogo (F), Brava (Br) (Southern Group).

(3) Feijó collections with a * refers to specimens in Herbaria; otherwise the references were reported in handwritten manuscripts of Feijó (1783-1789 - Plant collections and manuscripts; 1788 - Plantae Insulanae; see Gardére et al. 2019).

a Some of the previous references to *Phoenix dactylifera* (e.g. Feijó 1797, Smith 1818, Schmidt 1852) are probably related with *Phoenix atlantica*, since the two species (as well as hybrids) co-occur in the islands.

b Feijó collection [referred as Mus. Reg.Paris in Niger Flora: 98] is not reported in Gardére et al. 2019.

Table S2. Natural characteristics of the islands. TRI stands for Terrain Ruggedness Index, a measure of the ruggedness of an island (see main text for details) and SIE for "Single Island Endemics".

Island	Area (km^2)	Altitude (m)	Age (mya)	TRI	Endemic Species	SIE
Brava	62.51	976	3	27.21	27	1
Boavista	631.1	387	10	4.746	16	0
Fogo	476	2,829	3	22.46	40	6
Maio	274.5	436	12	4.151	13	0
Sal	219.8	406	15.8	3.278	15	1
Santo Antão	779	1,979	3	32.08	51	11
São Nicolau	343	1,312	4.7	21.92	49	10
Santiago	991	1,392	6	18.67	40	3
São Vicente	226.7	744	4.43	15.15	39	2

Table S3. List of the main collectors and periods they stayed in Cabo Verde Islands, until the first half of the 20th century.

Date	Collector	Abbreviation
**17th and 18th centuries**		
1699	Willem Adriaan van der Stel*	
1772?	Robertson, James	Robertson
1772	Forster, Johann Reinhold & Forster, Georg	J.R. Forster & Forster
1783-1796	Feijó, João da Silva	Feijó
1792	Staunton, George Leonard	D.A.Sutton
**19th century**		
1816	Leschenault, Jean	Leschenault
1816	Smith, Christen	Smith
1819	Perrottet, Georges	Perrottet
1820	Saint-Hilaire, Auguste	Saint-Hilaire
1822	Forbes, John	Forbes
1823	Bowdich, Sara	Bowdich
1823	Bowdich, Thomas. E.	T.E. Bowdich
1824	Jussieu, Adrien	Jussieu
1824, 1829	Leprieur, F. R.	Leprieur
1832	Darwin, Charles	Darwin
1835, 1837	Heudelot	Heudelot
1838, 1839	Brunner, Samuel	Brunner
1839	Hooker, Joseph Dalton	Hooker
1841	Vogel, J.R. Theodor	Vogel
1842	Afzelius, Adam	Afzelius
1846	Pabst, Carl	Pabst
1850	Bocandé, Bertrand: Santiago	Bocandé
1851	Schmidt, Johan Anton	Schmidt
1851-52, 1853, 1854	Bolle, Carl	Bolle
1852	MacGillivray, John	MacGillivray
1853, 1861	Welwitsch, Friedrich	Welwitsch
1859, 1860	Maly	Maly
1859, 1860	Wawra von Fernsee, H.R.	Wawra
1861	Torres, I.	Torres
1862, 1864, 1865-1866	Lowe, Richard Thomas	Lowe
1865, 1879, 1884-85, 1889	Krause, E.H.L.	Krause
1866	Stubel	Stubel
1866	Wollaston, V.	Wollaston
1873	Hopffer, F.F.	Hopffer
1873	Moseley, H.N.	Moseley
1873, 1883	Balansa, B.	Balansa
1874	Naumann, M.	Naumann

1876	Savatier, P.A.Lud	Savatier
1881, 1900	Newton, Francisco	Newton
1882	Loreno, A.	Loreno
1882	Marcacci, M.	Marcacci
1883	Poirault, Georges	Poirault
1883-1905	Cardoso Junior, João A.	Cardoso
1894-1922, 1934	Dinklage, Max Julius	Dinklage
1897-98	Fea, Leonardo	Fea
**First half of 20th century**		
1903	Werth, E.	Werth
1904	Freitas, A. Barjona de	Freitas
1908	Andrade, A. da Costa: Missão Agronómica a Cabo Verde	Andrade
1908	Capitão, Pereira da Cunha: Missão Agronómica a Cabo Verde	Capitão
1908	Costa Andrade, A. da (ver Andrade)	Andrade
1908	Cunha Capitão, Pereira da (ver Capitão)	Capitão
1908	Lemos, C. Pinto de: Missão Agronómica de Cabo Verde	Lemos
1908-1909	Lemos, Pinto de; Capitão, Pereira da Cunha & Andrade, A. Da Costa: Missão Agronómica a Cabo Verde	M. E. A.
1929	Missão Técnica de Arborização	M. T. A.
1933-1934	Baptista, Manuel Martins	Baptista
1934	Chevalier, Auguste	Chevalier
1934, 1935	Nobre	Nobre
1935	Santo, J. Espirito	Espirito Santo
1938	Barbosa, Ilídio	I. Barbosa
1938	Gossweiler, John	Gossweiler
1947-1948	Berhaut, Jean	Berhaut
1953-54	Lindberg, H	Lindberg
1955-56, 1961, 1982-83	Barbosa, Luís A. Grandvaux	Barbosa

Notes.
*****Willem Adriaan van der Stel visited Santiago in 1699 en route to the Cape, and he sent seeds of *Lotus jacobaeus* to the Botanic Garden of Amsterdam.

Figures:

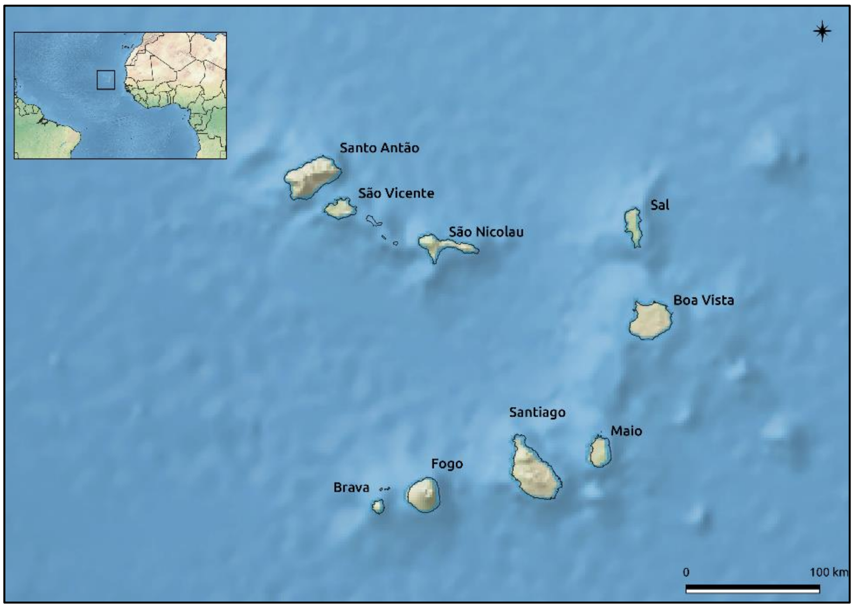

Figure S1. The Cabo Verde archipelago

Complements to the Bayesian analysis

The explanatory variables

We tested a variety of models, but here we discussed only the subset that led to the lowest WAIC values and the best fits; all included the Gaussian process. It is worth first remarking on a few characteristics of the relationships between the several variables considered: age of the island, maximum altitude, area and TRI. These variables are likely to impact the total number of species, in particular the number of endemic species found in an island, but they also may hinder the collection of species. As **Figures S2** and **S3** show, they exhibit patterns that are very interesting to explore, but such an enterprise is clearly outside the scope of the present work. What is relevant for our purposes is that with exception of area, these variables are strongly correlated. This can be observed in **Figure S2** where we show the linear values and in **Figure S3** where we show their logarithmic values. Notice in the latter figure the almost perfectly linear relationships between the logarithm of the age and of TRI, revealing a potential power law relationship. After several tests, and in order to avoid correlations among the variables, we concluded the best models were those that included the spatial autocorrelation term, the presence or absence of the harbour and an additional explanatory variable.

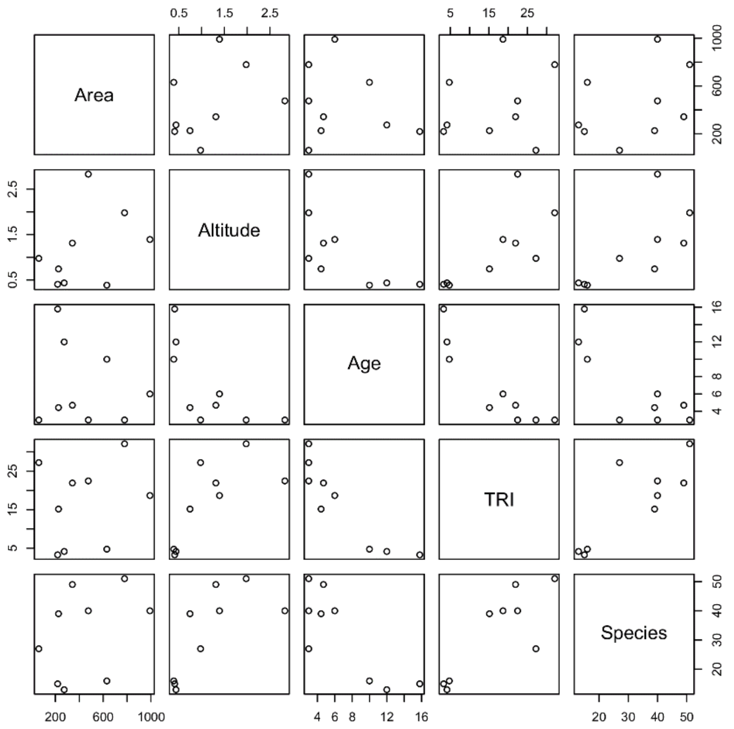

Figure S2. The relationship between the linear values of age of the islands, their maximum altitude, area and TRI. All the values are in arbitrary unities.

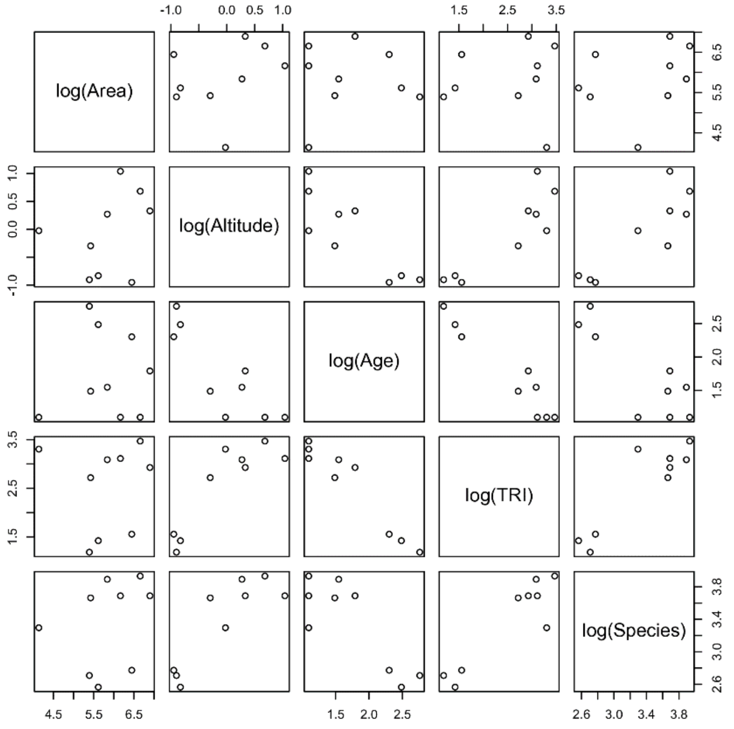

Figure S3. The relationship between the logarithmic values of age of the islands, their maximum altitude, area and TRI. All the values are in arbitrary unities.

The basic model

We used the package "Rethinking" developed by McElreath (2015) that offers the function "map2stan" which provides a simpler way of coding a model than the original Stan package. An example of our model using the TRI variable is (see details in McElreath (2015), chapter 13):

```
m.ah.ac <- map2stan(
  alist(
    Spp_col ~ dbinom(lambda),
    log(lambda) <- a + ba*TRI_s + bh*Harbour + g[Isl_code],
    g[Isl_code] ~ GPL2( Dmat , etasq , rhosq , 0.01 ),
    a  ~ normal( 0 , 10 ),
    ba ~ normal( 0 , 10 ),
    bh ~ normal( 0 , 10 ),
    etasq ~ dcauchy(0,1),
    rhosq ~ dcauchy(0,1)
  ),
  data=list(Spp_col=d$Spp_col, TRI_s=d$TRI_s, Harbour=d$Harbour,
          Isl_code=d$Isl_code, Dmat=Dmat),
  warmup=2000, iter=12000, chains=2)
```

Notice the index "_s" which means that the values of the variables have been standardized, that is, given a variable *x* we first subtracted its mean and then divided by its standard deviation, which is then *x*_s..

Detailed results

Covariance

The covariance, Γ,

$$\Gamma\ = \eta^2 \exp(-\rho^2 D^2) + 0.01\ \delta_{ij}$$

estimated from sampling the posteriors of η^2 and ρ^2, for several values of distance, *D*, is shown in **Figure S4** for the six years studied, together with their median. Observe that independently of the year the median decays to almost zero after 200km and that most samples are almost equal to zero after 400km.

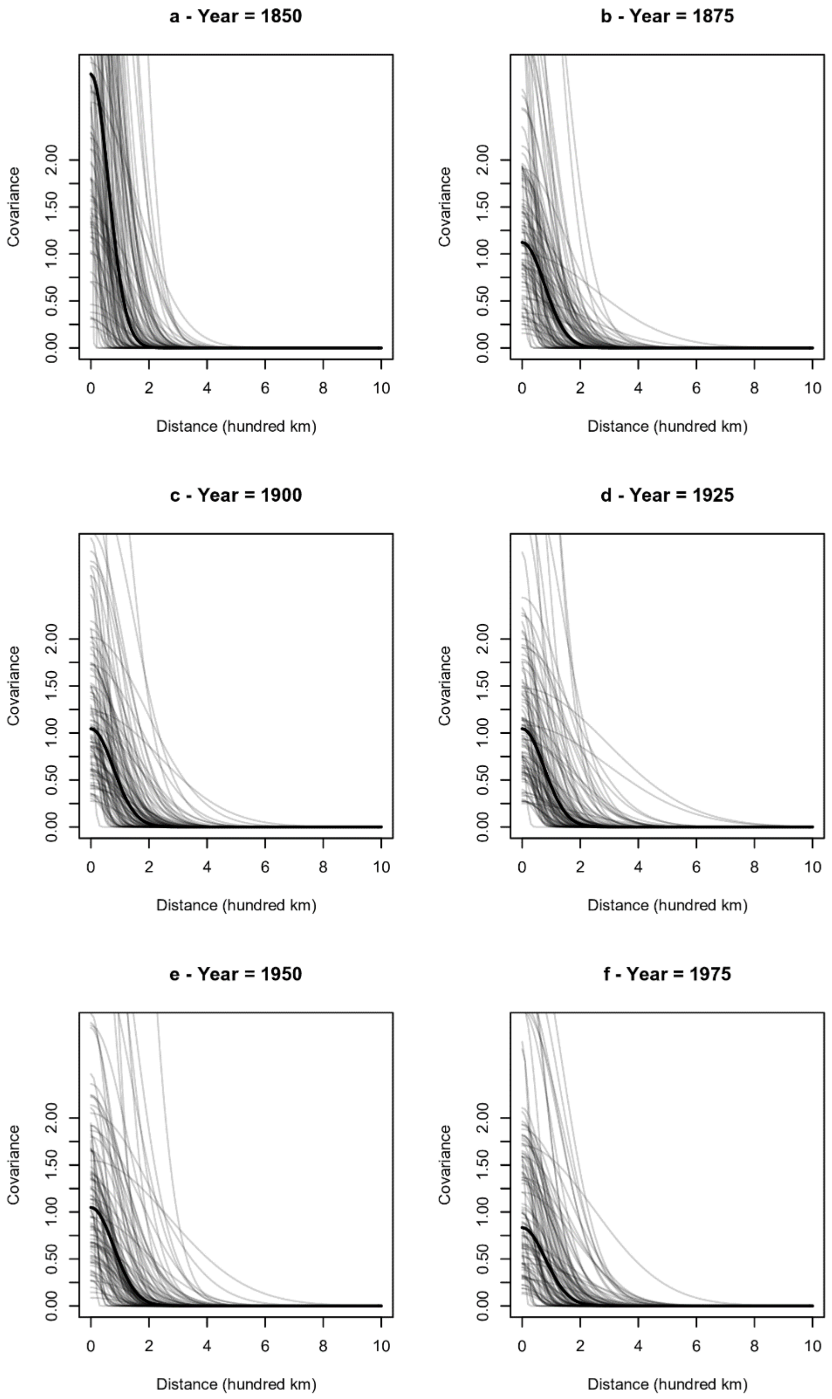

Figure S4. One hundred sampled values of the covariance sampled from the posterior of η^2 and ρ^2, for several values of distance (grey lines), for the six years studied. The black line corresponds to the median value of 10,000 samples.

Depicting correlations in geographical space

In the main text we showed the correlations in geographical space for only 1850 and 1975, **Figure 4.** Here, for completeness we show the results for all years, **Figure S5.**

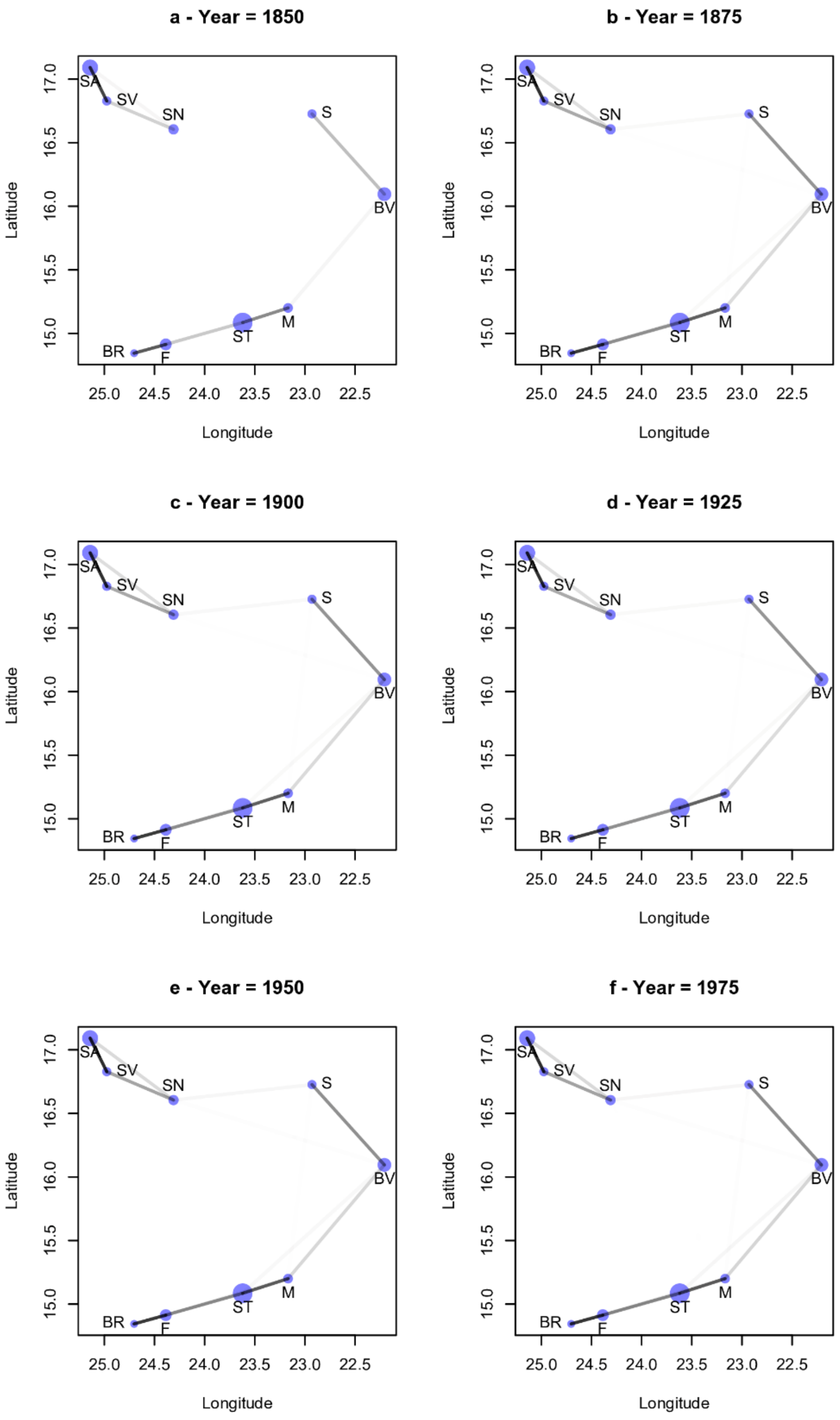

Figure S5. The dots correspond to the location of the islands in geographic space, and the size of the circles are proportional to the size of the islands. The darker the lines between the islands the larger the corresponding value in the correlation matrix (see main text). All pairs of islands have lines connecting them, however, in some cases the correlation values are so low that the lines are almost white, and thus very difficult to discern. Notice that the plots are very similar to all years. Island abbreviations: Santo Antão (SA), São Vicente (SV), São Nicolau (SN); Sal (S), Boavista (BV), Maio (M); Santiago (ST), Fogo (F), Brava (Br).

Finally, in **Figure S6** we show the number of collected species as a function of TRI for all years.

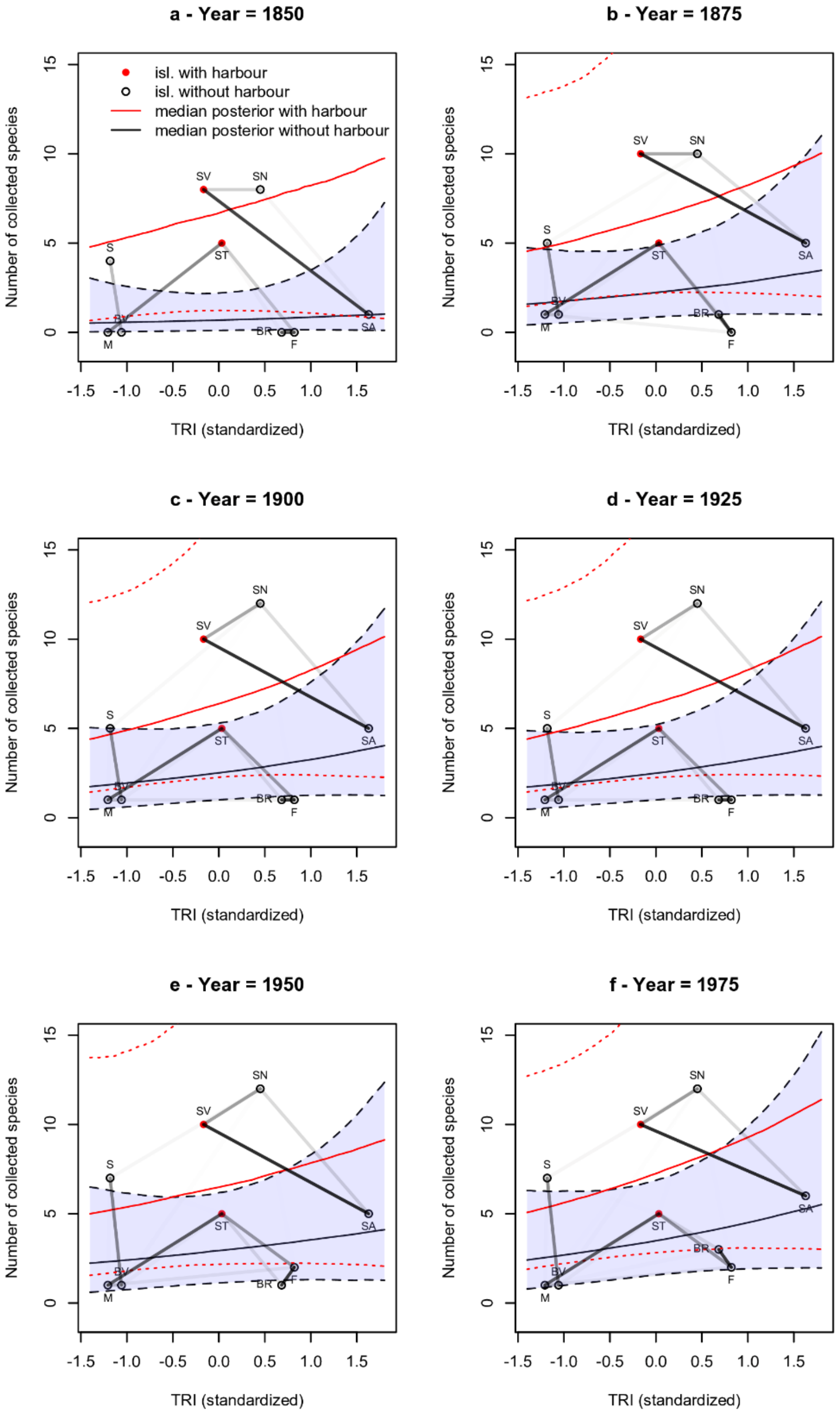

Figure S6. The number of collected species as a function of (standardized) TRI for all time periods, plots a-f. The values corresponding to islands with harbours (Santiago and São Vicente) are shown with red dots, the others are in black. The dotted line represents the mean of the average posterior predictive relationship between TRI and the proportion of species collected. The dotted line corresponds to the median of the posterior for the islands with a harbour and the dashed line to the median of the posterior for islands without harbours. The grey shade denotes the 80% confidence intervals of the posterior of

the islands with a major harbour and the blue shade the 80% confidence intervals of the posterior of the islands without a major harbour; where these two shaded regions intercept there is a darker shaded region. To these results we superimposed the values of correlations of the spatial autocorrelation between the islands with darker lines corresponding to stronger correlations. Island abbreviations: Santo Antão (SA), São Vicente (SV), São Nicolau (SN); Sal (S), Boavista (BV), Maio (M); Santiago (ST), Fogo (F), Brava (BR).
